# Effect of prone position in patients with acute respiratory distress syndrome supported by venovenous extracorporeal membrane oxygenation: a retrospective cohort study

**DOI:** 10.1186/s12890-022-02026-7

**Published:** 2022-06-16

**Authors:** Ziying Chen, Min Li, Sichao Gu, Xu Huang, Jingen Xia, Qinghua Ye, Jiangnan Zheng, Qingyuan Zhan, Chen Wang

**Affiliations:** 1 Peking University China-Japan Friendship School of Clinical Medicine, No. 2 Yinghua East Road, Chaoyang District, Beijing, 100029 People’s Republic of China; 2 Center for Respiratory Diseases, China-Japan Friendship Hospital, Beijing, People’s Republic of China; 3 Department of Pulmonary and Critical Care Medicine, China-Japan Friendship Hospital, Beijing, People’s Republic of China; 4 National Clinical Research Center for Respiratory Diseases, Beijing, People’s Republic of China; 5 Chinese Academy of Medical Sciences and Peking Union Medical College, Beijing, People’s Republic of China; 6 Department of Pulmonary and Critical Care Medicine, Suzhou Ninth Hospital Affiliated to Soochow University, Suzhou, People’s Republic of China

**Keywords:** Acute respiratory distress syndrome, Prone position, Venovenous extracorporeal membrane oxygenation

## Abstract

**Background:**

The application of prone position (PP) in acute respiratory distress syndrome (ARDS) supported by venovenous extracorporeal membrane oxygenation (VV-ECMO) is controversial.

**Objectives:**

To evaluate the safety and efficacy of application of PP during VV-ECMO in patients with ARDS.

**Methods:**

This was a single-center, retrospective study of patients who met the Berlin definition of ARDS, and were supported with VV-ECMO. We divided the patients into two groups. The prone group included patients who were supported by VV-ECMO, and experienced at least one period of PP, while those without PP during VV-ECMO were defined as the supine group. Propensity score matching (PSM) at a ratio of 1:1 was introduced to minimize potential confounders. The primary outcomes were the complications of PP and the change of arterial oxygen pressure/fraction of the inspiration (PaO_2_/FiO_2_) ratio after PP. The secondary outcomes were hospital survival, ICU survival, and ECMO weaning rate.

**Results:**

From April 2013 to October 2020, a total of 91 patients met the diagnostic criteria of ARDS who were supported with ECMO. 38 patients (41.8%) received at least one period of PP during ECMO, while 53 patients (58.2%) were maintained in supine position during ECMO. 22 minor complications were reported in the prone group and major complications were not found. The other ECMO-related complications were similar between two groups. The PaO_2_/FiO_2_ ratio significantly improved after PP compared with before (174.50 (132.40–228.25) mmHg vs. 158.00 (122.93–210.33) mmHg, *p* < 0.001). PSM selected 25 pairs of patients with similar characteristics. Hospital survival or ICU survival did not differ between the two groups (40% vs. 28%, *p* = 0.370; 40% vs. 32%, *p* = 0.556). Significant difference of ECMO weaning rate between two groups was not found (56% vs. 32%, *p* = 0.087).

**Conclusions:**

PP during VV-ECMO was safe and could improve oxygenation. A large-scale and well-designed RCT is needed in the future.

**Supplementary Information:**

The online version contains supplementary material available at 10.1186/s12890-022-02026-7.

## Introduction

Acute respiratory distress syndrome (ARDS) is a type of acute, diffuse, inflammatory lung injury that is characterized by the rapid development of hypoxemia, and is associated with high mortality and high health care costs especially in the intensive care unit (ICU) [1–3]. Prone position (PP) as a postural therapy can optimize respiratory mechanics, improve ventilation-perfusion matching, ameliorate the right ventricular function, and reduce the risk of ventilator induced lung injury (VILI) via reduction of the stress and strain across the lungs [4–8]. The Positioning Severe ARDS Patients (PROSEVA) study demonstrated that early and prolonged mechanical ventilation in PP was associated with significant survival advantage for patients with severe ARDS [9]. As a potent salvage therapy, venovenous extracorporeal membrane oxygenation (VV-ECMO) rests the lungs and reduces the risk of VILI for severe patients with ARDS [10]. The individual participant data meta-analysis of the ECMO to Rescue Lung Injury in Severe ARDS (EOLIA) and the Conventional Ventilatory Support versus Extracorporeal Membrane Oxygenation for Severe Adult Respiratory Failure (CERSA) trials supported the efficacy of ECMO for patients with ARDS in improving survival rate [11].

The above studies demonstrated better prognoses of patients with ARDS who were treated by ECMO or PP, while the safety and efficacy of combination of these two therapies has not been evaluated. In clinical practice, the use of PP in patients with ARDS supported by ECMO is limited by lack of experience and potential risk of serious complications, such as bleeding from cannula insertion sites, dislodgment, and decrease of extracorporeal blood flow or extreme hemodynamic instability etc. Previous studies reported that PP during ECMO was safe and improved oxygenation [12–14]. However, in recent years, efficacy evaluation tended to focus on survival rate and ECMO weaning rate, and the results of studies were controversial [15–25].

Thus, we performed a retrospective analysis of PP in patients with ARDS supported by ECMO, which aimed to evaluate the safety and efficacy. The primary outcomes were the safety of PP during ECMO and the change of arterial oxygen pressure/fraction of inspiration (PaO_2_/FiO_2_) ratio after PP. The secondary outcomes were hospital survival, ICU survival, and ECMO weaning rate.

## Methods

### Study design and population

This is a retrospective study enrolling patients treated in the 30-bed medical intensive care unit (MICU) of a teaching hospital at Beijing, China from April 2013 to October 2020. All 91 patients included in our study met the Berlin definition of ARDS [2] and were supported with VV-ECMO. All participants were older than 14 years because otherwise they would be triaged to pediatric department. We did not include patients with COVID-19. Patients who received at least one period of PP during ECMO were categorized into the prone group, while those without PP were defined as the supine group. ARDS treatment was carried out in both groups according to the currently valid guidelines.

Patients’ demographic characteristics, comorbidities, risk factors, pathogen, laboratory examinations, organ support, and ventilation or ECMO parameters were obtained from the hospital electronic medical records system, nursing records system, intensive care system, laboratory examination system, and radiological examination system.

First, we compared the two groups to explore the differences at the time of admission, before ECMO, and the first day of ECMO. Propensity score matching (PSM) at a ratio of 1:1 was introduced to minimize influence of potential confounders. Second, in the prone group, an analysis was conducted by comparing ventilation, ECMO, and arterial blood gas parameters between before and after prone position.

We have attached the STROBE guidelines as a supplementary table to ensure adequate quality of reporting of the study (Additional file 1: Table S10).

### VV-ECMO and prone position management

#### VV-ECMO

VV-ECMO was performed using a venovenous circuit with two cannulas, usually in the femoral vein and internal jugular vein. Cannulations were performed by two experienced physicians in Seldinger’s technique, and all circuit surfaces were heparin-coated. The goal of anticoagulation was individualized according to risk of bleeding, coagulant function, and thrombotic tendency, which was monitored by measuring activated clotting time (ACT) and activated partial prothrombin time (APTT) every 4 h. In order to minimize the occurrence of VILI, the parameters of mechanical ventilation (MV) was reduced during ECMO, and the ECMO blood flow was adjusted to maintain the peripheral oxygen saturation level of 85–90%, and the partial pressure of arterial oxygen level of 50 mmHg. Typical ventilator settings were as follows: respiratory rate ≤ 25 bpm, tidal volume ≤ 4–6 ml/kg, positive end expiratory pressure (PEEP) ≤ 15cmH_2_O, plateau pressure ≤ 30cmH_2_O, driving pressure ≤ 15cmH_2_O, and FiO_2_ ≤ 0.5. The management of fluid balance and the use of vasoactive drugs were driven by clinical judgement of physicians. Sedative and analgesic drugs were routinely used to achieve a RASS (Richmond Agitation Sedation Scale) between − 3 and − 1, and unless patients had high-risk of lung injury or strong respiratory drive, neuromuscular blocking agents were not routinely given. All cannulas and lines were carefully handled, and the running status of VV-ECMO was closely monitored by medical team. ECMO weaning, which is defined as being free from ECMO and alive for at least 48 h, was attempted after the patient has improved sufficiently with reasonable ventilator settings such as FiO_2_ < 0.4, peak inspiratory pressure (PIP) < 25cmH_2_O, stable breathing pattern, and respiration rate < 30 bpm.

#### Prone position

According to the judgment of our medical team, PP was done in patients who had difficulties in maintenance of oxygenation, requirement of aggressive sputum drainage, or had potential difficulties in weaning ECMO. When PP was performed in ECMO patients, at least six health care workers were involved, including four (usually physicians) performing the turning of the patient, one (usually a nurse) looking after the ECMO and other circuit, and one (usually a respiratory therapist) for the management and protection of the endotracheal tube. Usually, the duration of one period of PP was between 12 and 16 h, unless the occurrence of major irreversible or life-threatening complication.

### Complications

Complications were classified into major (cardiac arrest, difficult to correct hemodynamic instability, malignant arrhythmia, or dislodgment) and minor (pressure sores, edema, vomiting, or other reversible complications). It is noteworthy that the dislodgement of ECMO cannulas, which could lead to irreversible decrease of blood flow on ECMO and need adjust the position of ECMO cannulas, was considered a major complication.

### Statistical analysis

Statistical analysis was conducted with SPSS 26.0 for Windows software (SPSS Inc. Chicago. IL. USA). A statistical significance was defined as a *p-value* less than 0.05. Categorical variables were reported as frequency and percentage, continuous variables were reported as mean (standard deviation) or medians (interquartile ranges). Categorical variables were compared using Chi-square or Fisher’s exact test, and for continuous variables, the *t* test or Mann–Whitney *U* test or Wilcoxon rank sum test was used to assess the differences between groups.

PSM method was applied at ratio of 1:1 between the prone group and the supine group, with a nearest neighbor matching algorithm using a caliper of 0.2. Age, pathogen spectrum, respiratory support, PaO_2_/FiO_2_ ratio prior to ECMO, prone position before ECMO, and barotrauma before and during ECMO were matched in PSM to derive the cohort.

## Results

### Baseline characteristics

**Fig. 1:**
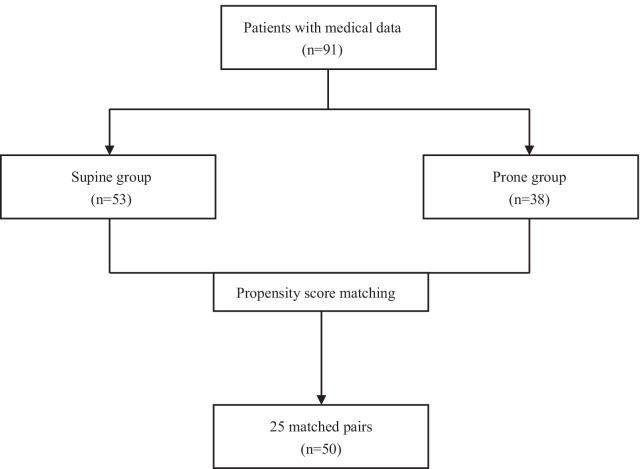
A flowchart illustrated the enrollment of patients in our study. Propensity score matching was performed for age, pathogen spectrum, respiratory support, PaO_2_/FiO_2_ ratio prior to ECMO, PP before ECMO, barotrauma before and during ECMO. ECMO, extracorporeal membrane oxygenation; MV, mechanical ventilation; PP, prone positioning

**Fig. 2:**
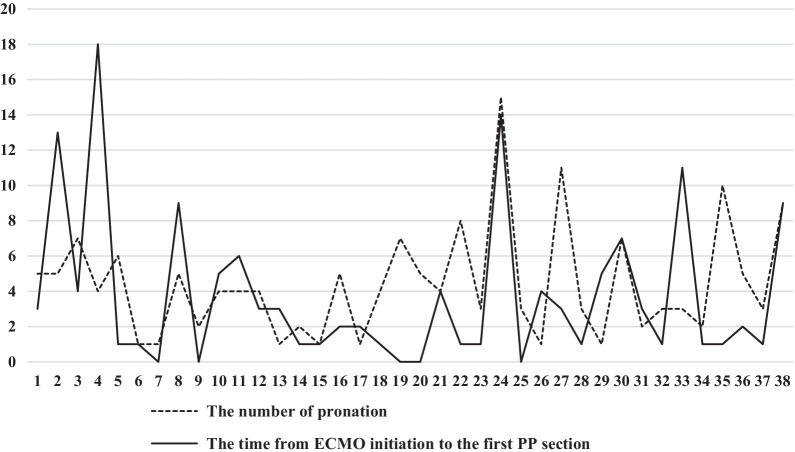
The number of pronation for patients and the time from ECMO initiation to the first prone position section

38 patients (41.8%) received at least one period of PP during VV-ECMO, while 53 patients (58.2%) were maintained in supine position during VV-ECMO (Fig. 1). A total of 167 periods of PP were performed throughout the study. The mean duration of PP was 14 (12–16) h. The time from ECMO initiation to the first PP section was 2 (0.9–4.9) days. 4 (2.0–5.3) PP episodes were performed per patient (Additional file 1: Table S9, Fig. 2). The mean age of all patients was 49 (37–63) years old, male patients accounted for 70.3% (n = 64), and the mean Sequential Organ Failure Assessment (SOFA) score was 9 (6–12) points. PSM selected 25 pairs of patients with similar characteristics (Table 1).

**Table 1:** Baseline characteristic of the entire cohort and after propensity score matching analysis

	Not propensity score matched				Propensity score matched		
	Entire cohort (n = 91)	Prone group (n = 38)	Supine group (n = 53)	*p*	Prone group (n = 25)	Supine group (n = 25)	*p*
Males [n (%)]	64 (70.3)	29 (76.3)	35 (66)	0.29	18 (72)	16 (64)	0.544
Age (years)	49 (37–63)	41 (31–50)	58 (44–64)	0.001^b^	46.68 ± 13.42	48.36 ± 13.29	0.658
BMI (kg/m^2^)	24.77 (22.03–28.27)	25.25 (22.25–28.54)	24.6 (20.94–28.09)	0.566	25.85 ± 3.99	26.24 ± 5.88	0.785
Cause of ARDS [n (%)]							
Bacteria	20 (22)	5 (13.2)	15 (28.3)	0.113	4 (16)	8 (32)	0.185
Fungal	6 (6.6)	3 (7.9)	3 (5.7)	0.536	1 (4)	1 (4)	1
PCP	15 (16.5)	4 (10.5)	11 (20.8)	0.228	4 (16)	3 (12)	0.684
Viral	57 (62.6)	32 (84.2)	25 (47.2)	0.001^b^	20 (80)	16 (64)	0.208
Influenza virus	43 (47.3)	24 (63.2)	19 (35.9)	0.010^a^	13 (52)	13 (52)	1
Comorbidities [n (%)]							
Hypertension	17 (34)	8 (32)	9 (36)	0.765	8 (32)	9 (36)	0.765
Diabetes mellitus	10 (20)	7 (28)	3 (12)	0.157	7 (28)	3 (12)	0.157
Chronic heart failure	1 (2)	0	1 (4)	1	0	1 (4)	1
Chronic renal disease	1 (2)	0	1 (4)	1	0	1 (4)	1
COPD	0	0	0		0	0	
Asthma	2 (4)	1 (4)	1 (1)	1	1 (4)	1 (1)	1
Tuberculosis	1 (2)	0 (0)	1 (4)	1	0 (0)	1 (4)	1
Chronic liver disease/cirrhosis	2 (4)	0 (0)	2 (8)	0.490	0 (0)	2 (8)	0.490
Immunocompromised status	14 (28)	7 (28)	7 (28)	1	7 (28)	7 (28)	1
Cerebrovascular disease	3 (6)	2 (8)	1 (4)	1	2 (8)	1 (4)	1
Smoking [n (%)]	54 (59.3)	24 (63.2)	30 (56.6)	0.530	14 (56)	16 (64)	0.564
Alcohol [n (%)]	14 (15.4)	7 (18.4)	7 (13.2)	0.317	5 (20)	4 (16)	0.546
MV before ECMO (h)	48 (11–120)	55.5 (20–132)	45 (7.5–120)	0.291	49 (22–144)	62 (16.5–120)	0.596
PP before ECMO [n (%)]	31 (34.1)	14 (26.4)	17 (44.7)	0.069	11 (44)	7 (28)	0.239
Before ECMO							
IPPV	82 (90.1)	38 (100)	44 (83)	0.009^b^	25 (100)	25 (100)	1
Barotrauma [n (%)]	18 (19.8)	12 (31.6)	6 (11.3)	0.017^a^	6 (24)	4 (16)	0.480
PaO_2_/FiO_2_ (mmHg)	64.9 (56–88.7)	65.9 (55.88–82.60)	63.3 (56–91.75)	0.449	65.70 (60.60–84.00)	63 (48.65–95.35)	0.810
Fluid balance (mL)	0 (− 3653 to 2060)	190 (− 4080 to 2119)	0 (− 3593 to 2589)	0.705	381 (− 2625 to 1799)	0 (− 3894 to 4769)	0.938

ECMO, extracorporeal membrane oxygenation; ICU, intensive care unit; MV, mechanical ventilation; IPPV, invasive positive pressure ventilation

^a^*p* < 0.05; ^b^*p* < 0.01

Before matching, we found statistical significance in age and the pathogen spectrum of ARDS induced by pneumonia. The prone group had a median age of 41 (31–50) years old, compared to 58 (44–64) years old in the supine group (*p* = 0.001). Viral infection, especially influenza virus, was the main pathogen of ARDS (62.6%), and was more common in the prone group (84.2% vs. 47.2%, *p* = 0.001). Invasive positive pressure ventilation (IPPV) before ECMO was widely used in patients (90.1%), and moreover, all patients in the prone group were supported by IPPV. Barotrauma before ECMO was more common in the prone group than the supine group (31.6% vs. 11.3%, *p* = 0.017). In addition, the prone group had a higher level of total bilirubin (TBIL), creatine kinase (CK), and brain natriuretic peptide (BNP) than the supine group. After matching, all baseline characteristics and parameters before ECMO and on the first day of ECMO were similar (Table 1, Additional file 1: Table S1–S4).

### Primary and secondary outcomes

#### Complications

In the prone group, 16 patients suffered from at least one complication, 22 minor complications were reported totally, which were considered as prone positioning complications. Pressure sores and edema were observed in several patients. And a decrease in systolic blood pressure (SBP) was encountered in one patient, which was subsequently recovered by fluid infusion and vasoactive agents. Beyond that, in seven patients we found a decrease of blood flow on ECMO, which was resolved by fluid infusion or optimized positioning in PP. Besides, we also found a increase of PaCO_2_ in one patient and a decrease of SpO_2_ in another, which were both subsequently corrected by the adjustment of ECMO and the ventilator parameters. Major complications such as dislodgment or difficult to correct hemodynamic instability or malignant ventricular arrhythmia or severe bleeding were not found (Table 2). The occurrence of ECMO-related complications were similar between the two groups (Additional file 1: Table S5).

**Table 2:** Complications associated with prone position

Complication	Prone group (n = 38)
Pressure sores (Face)	4 (10%)
Edema	6 (15%)
Vomiting	1 (2%)
Minimal bleeding	3 (7%)
Increase of CO_2_	1 (2%)
Decrease of SpO_2_	6 (15%)
Decrease of systolic blood pressure	1 (2%)
Decrease of blood flow on ECMO	7 (18%)
Dislodgment	0
Severe bleeding	0
Difficult to correct hemodynamic instability	0
Malignant ventricular arrhythmia	0

CO_2_, carbon dioxide; SpO_2_, peripheral oxygen saturation; ECMO, Extracorporeal Membrane Oxygenation

#### The change of PaO_2_/FiO_2_ ratio

The PaO_2_/FiO_2_ ratio significantly improved after PP compared with before (174.50 (132.40–228.25) mmHg vs. 158.00 (122.93–210.33) mmHg, *p* < 0.001). While heart rate (HR) (103 ± 22 vs. 98 ± 22 bpm, *p* = 0.003), SBP (131 ± 20 vs. 125 ± 21 mmHg, *p* < 0.001) and mean arterial pressure (MAP) (86 ± 13 vs. 83 ± 13 mmHg, *p* = 0.016) were lower after PP. Furthermore, tidal volume significantly decreased after PP (7.48 (5.43–10.70) ml/kg vs. 7.01 (5.23–8.95) ml/kg, *p* = 0.009). We also found the blood flow of ECMO was decreased during PP (4.09 ± 0.75 vs. 3.98 ± 0.82, *p* < 0.001) (Table 3).

**Table 3:** Ventilator, ECMO and arterial blood gas parameters before and after prone positioning in the prone group

	Before prone	After prone	*p*
Heart rate (beats/min)	103 ± 22	98 ± 22	0.003^b^
Systolic pressure (mmHg)	131 ± 20	125 ± 21	< 0.001^b^
Diastolic pressure (mmHg)	63 ± 12	63 ± 12	0.429
Mean arterial pressure (mmHg)	86 ± 13	83 ± 13	0.016^a^
Blood flow (L/min)	4.09 ± 0.75	3.98 ± 0.82	< 0.001^b^
FiO_2_ of ECMO	1	1	1
FiO_2_ of ventilator	0.45 (0.40–0.55)	0.45 (0.40–0.50)	0.004^b^
Tidal volume (mL/kg)	7.48 (5.43–10.70)	7.01 (5.23–8.95)	0.009^b^
Plateau pressure (cmH_2_O)	22 (19–25)	22 (19–24)	0.360
PEEP (cmH_2_O)	10 ± 3	10 ± 3	0.546
Respiratory rate (breaths/min)	21 (15–28)	20 (15–26)	0.359
pH	7.41 ± 0.06	7.41 ± 0.057	0.131
PaO_2_ (mmHg)	73.20 (63.75–86.40)	76.40 (64.60–91.40)	0.009^b^
PaO_2_/FiO_2_ (mmHg)	158.00 (122.93–210.33)	174.50 (132.40–228.25)	< 0.001^b^
PaCO_2_ (mmHg)	44.50 (10.90–47.75)	43.80 (40.40–47.40)	0.132
Lac (mmol/L)	1.9 (1.4–2.6)	1.7 (1.3–2.6)	0.495

PEEP, positive end expiratory pressure; pH, potential of hydrogen; PaO_2_, arterial oxygen pressure; FiO_2_, fraction of inspiration; PaCO_2_, partial pressure of arterial carbon dioxide; Lac, lactic acid; ECMO, extracorporeal membrane oxygenation

^a^*p* < 0.05; ^b^*p* < 0.01

#### Survival rate and ECMO weaning

In the PSM cohort, there was no statistically significant difference in hospital or ICU survival between the prone group and the supine group (40% vs. 28%, *p* = 0.370; 40% vs. 32%, *p* = 0.556). In addition, the difference in ECMO weaning rate between the two groups was also insignificant (56% vs. 32%, *p* = 0.087) (Table 4). To test the stability of the results, we also included these in logistic regression model (Additional file 1: Table S6–S8). Patients in the prone group had a longer duration of ECMO support than the supine group (14.5 (9.25–29.75) d vs. 8.00 (3.25–16) d, *p* = 0.007). Besides, the length of hospital stay (16 (8–27) d vs. 30 (17–40.5) d, *p* = 0.018) and the length of ICU stay (14 (6.69–25.94) d vs. 28.33 (16–40.5) d, *p* = 0.014) were also longer in the prone group (Table 4).

**Table 4:** Outcomes for patients in two groups

	Not propensity score matched				Propensity score matched		
	Entire cohort (n = 91)	Prone group (n = 38)	Supine group (n = 53)	*p*	Prone group (n = 25)	Supine group (n = 25)	*p*
Organ failure [n(%)]							
Renal	47 (54)	22 (57.9)	25 (51)	0.523	15 (60)	15 (56)	0.774
Cardiovascular	52 (58.4)	19 (51.4)	33 (63.5)	0.253	13 (52)	18 (72)	0.145
Hepatic	26 (29.6)	10 (27)	16 (31.4)	0.659	8 (32)	8 (32)	1
Coagulation	17 (18.9)	7 (18.9)	10 (19.6)	0.936	6 (24)	3 (12)	0.269
Central nervous system	21 (24.1)	8 (21.6)	13 (26.0)	0.637	6 (24)	9 (37.5)	0.305
≥ 1 organ failure	67 (73.6)	27 (71.1)	40 (75.5)	0.637	19 (76)	20 (80)	0.733
≥ 2 organs failure	43 (47.3)	18 (47.4)	25 (47.2)	0.985	14 (56)	14 (56)	1
ICU survival [n (%)]	33 (36.3)	16 (42.1)	17 (32.1)	0.326	10 (40)	8 (32)	0.556
Hospital survival [n (%)]	29 (31.9)	16 (42.1)	13 (24.5)	0.076	10 (40)	7 (28)	0.370
ECMO weaning [n (%)]	38 (41.8)	20 (52.6)	18 (47.4)	0.075	14 (56)	8 (32)	0.087
Treatment abandoning [n (%)]	24 (26.4)	7 (18.4)	17 (32.1)	0.145	3 (12.5)	7 (28)	0.289
Length of ICU stay (d)	19 (9.8–35)	28.7 (17–47)	14 (5.7–23.5)	< 0.001^b^	28.33 (16–40.5)	14 (6.69–25.94)	0.014^a^
Length of hospital stay (d)	23 (11–38)	31.5 (17.5–59.75)	15 (6.5–26.5)	< 0.001^b^	30 (17–40.5)	16 (8–27)	0.018^a^
Duration of ECMO support (d)	10 (5–17)	14 (8.5–29.25)	8 (3–13)	0.001^b^	14.5 (9.25–29.75)	8 (3.25–16)	0.007^b^
MV duration (d)	13 (7–26.25)	19 (11.5–43.25)	11 (4.25–17.75)	0.002^b^	14 (12–43)	13 (7–20)	0.169

ECMO, Extracorporeal Membrane Oxygenation; ICU, Intensive Care Unit; MV, Mechanical Ventilation

^a^*p* < 0.05; ^b^*p* < 0.01

## Discussion

In this study, we explored the safety and efficacy of the application of PP in patients with ARDS supported with ECMO in a retrospective cohort. The main findings were of interest. First, PP in patients supported with VV-ECMO was safe. Second, the application of PP during VV-ECMO in patients with ARDS improved oxygenation without the enhancement of blood flow on ECMO or excess support of mechanical ventilation. However, there was no significant difference in hospital or ICU survival, or ECMO weaning rate.

We did not observe any major safety concerns such as severe bleeding from cannula insertion sites, dislodgment or extreme hemodynamic instability. However, owing to the design of retrospective analysis, some complications may be missed, but the major complications must have been documented in our study. All in all, in our study, there were no major complications in majority of the patients supported with ECMO. Therefore, with closely monitoring of the running status of VV-ECMO by experienced clinicians, PP during VV-ECMO is safe.

According to previous studies [12–14], there are consistent results indicating that PP during VV-ECMO in patients with ARDS improves oxygenation, although the time of assessment is not uniform. There are several potential explanations for the improvement of oxygenation of PP in patients with ARDS supported by VV-ECMO. First, the increased recruitment of alveolar in the dorsal lung results in better redistribution of ventilation in the dorsal regions where perfusion is maintained [26]. The better matching of the ventilation-perfusion ratio accounts for the improved oxygenation. Second, PP reduces stress and strain across the lungs, resulting in reduced risk of VILI [27]. Recently, Franchineau et al. used electric impedance tomography (EIT) to describe the impact of PP in patients receiving VV-ECMO and noted that the redistribution of tidal volumes and the end-expiratory lung impedance from the ventral to the dorsal regions with improvement were seen in static lung, which supports physiological benefits of PP in patients supported with ECMO, by modifying lung mechanics and potentially reducing risk of VILI [28]. In addition, PP can promote drainage of bronchial secretion and reduce right cardiac afterload [29]. However, oxygenation is affected by pump flow, recirculation of ECMO, heart minute output, and other comprehensive factors. Therefore, the results from our study are not able to confirm or negate other studies, owing to the limited number pf patients.

Our study did not show a significant difference in survival rate between the two groups, which was conflicting with previous studies [15, 16, 20, 24] and a meta-analysis of 13 studies [17]. Several reasons may explain this. First, in our center, we usually prefer to perform PP in patients who are difficult to maintain oxygenation after ECMO or have potential difficulties in weaning ECMO, while patients who have significant improvements in oxygenation after ECMO are usually maintained in supine position until ECMO weaning. It may generate an inter-relationship between PP and disease severity due to selection bias. The SOFA or APACHE II may not reflect the severity of lung disease. Second, we consider that the less duration of PP in our study results in the conflicting outcome (14 h in our study versus > 16 h in the PROSEVA trial). In addition, Zaaqoq et al. demonstrated that the duration of PP, time of day of PP, and the day of initiation of PP had temporal dimensions, which were unlikely to be dealt with properly in retrospective studies, might also contributed to bias [30]. Finally, our cohort was limited by a small sample size, even though prone positioning was used in younger patients with viral infections. Of note, patients in the prone group showed significantly prolonged duration of ECMO support and hospital stay. The first reason may be the survival advantage of PP, although the difference in survival rates is not statistically significant. Second, as mentioned above, patients in the prone group were more likely to have difficulties in maintenance of oxygenation after ECMO or potential difficulties in ECMO weaning. This phenomenon produces the immortal time bias. In short, patients need to be alive and event free to receive prone positioning, whereas others who died earlier on ECMO, or had ECMO stopped for futility did not receive prone positioning. Third, considering the higher treatment abandoning rate in the supine group, we deduced that patients in the supine group might have greater risk of organ failure or severity of illness, and thus shorten the duration of hospital stay. In addition, the need of more sedation and curarisation in prone group might increase the time of hospital stay.

Compared with the EOLIA study [31] and other major RCTs, our analysis reported a lower hospital or ICU survival, especially in the supine group. Several factors may account for this phenomenon. First, the PaO_2_/FiO_2_ was 64.9 (56–88.7) mmHg before ECMO in our analysis, and all patients met the criterion for ECMO treatment. Therefore, those patients who only met the mild or moderate ARDS criterion or only had high carbon dioxide levels were excluded. Second, the most common cause of ARDS in this study was pneumonia, which accounted for 96.7% and most of them had at least one organ failure (73.6%). This indicates the patients in our study have more severe illness. Last but not least, our center is considered as one of the major medical critical care centers in our country, and our patients are more likely to suffer higher risk of death who come from other regions. These above factors may contributed to the high mortality rate in our study.

In our study, we focused on the safety and efficacy of application of PP on patients with ARDS supported with ECMO. To our knowledge, this is the first analysis of the influence of PP in patients with ARDS who were supported with ECMO in China. However, this study has a number of limitations. First, this is a single-center retrospective study, the number of studied patients remains relatively small, and the criteria of deciding PP is uncertain, therefore possible selection bias and report bias are inevitable. Patients who had difficulty maintaining oxygenation may be eventually selected for PP, which may have contributed to the insignificant difference in survival rate between the two groups. Second, because of the differences between different centers, our findings may not be extended to other inexperienced centers. Besides, in the absence of randomization and standardization of mechanical ventilation and other treatment, it is not possible to quantify the true treatment effect. In addition, the time and the thresholds for initiating PP and duration of PP was not standardized. Finally, despite the applying of PSM, we cannot exclude residual confounding factors which may influence the results. To sum up, due to these limitations, a prospective randomized controlled study with larger sample size to explore the impact of PP on patients with ARDS supported by ECMO should be encouraged in the future.

## Conclusion

This retrospective analysis suggested PP during VV-ECMO was safe, and could improve oxygenation. A large-scale and well-designed RCT may provide more convincing evidence and is expected in the near future.

## Supplementary Information

**Additional file 1.** Supplementary tables.

## Data Availability

The datasets used and/or analysed during the current study are available from the corresponding author upon reasonable request.
