# Supplementary material for: Effect of prone position in patients with acute respiratory distress syndrome supported by venovenous extracorporeal membrane oxygenation: a retrospective cohort study
Source: BMC Pulm Med. 2022 Jun 16;22:234. doi: 10.1186/s12890-022-02026-7 (PMC9202669; doi:10.1186/s12890-022-02026-7)
Supplement: Supplementary file 1 — Additional file 1. Supplementary tables. [file 12890_2022_2026_MOESM1_ESM.docx]

Table S1. Before propensity score matching, laboratory examinations and respiratory support before ECMO in two groups

|  | **ALL**  **N=91** | **Prone group**  **N=38** | **Supine group**  **N=53** | **P** |
| --- | --- | --- | --- | --- |
| **Respiratory support, number (%)** | | | | |
| Nasal catheter/mask | 1 (1.1) | 0 (0) | 1 (1.9) | 1 |
| HFNC | 1 (1.1) | 0 (0) | 1 (1.9) | 1 |
| NPPV | 7 (7.7) | 0 (0) | 7 (13.2) | 0.039^a^ |
| IPPV | 82 (90.1) | 38 (100) | 44 (83) | 0.009^a^ |
| **Barotrauma before ECMO, number (%)** | 18 (19.8) | 12 (31.6) | 6 (11.3) | 0.017^a^ |
| **VAP, number (%)** | 10 (11) | 6 (15.8) | 4 (7.5) | 0.310 |
| **Tracheotomy, number (%)** | 59 (64.8) | 24 (63.2) | 35 (66) | 0.777 |
| **Laboratory examinations (IQR)** | | | | |
| WBC, *10^9/L | 10.89 (7.15-15.21) | 11.04 (5.68-15.14) | 10.52 (7.65-15.23) | 0.906 |
| LYM, *10^9/L | 0.57 (0.31-1.0) | 0.59 (0.33-0.91) | 0.56 (0.29-1.19) | 0.869 |
| HB, g/L | 107 (94.5-122) | 108 (95.25-124.25) | 106.5 (92.75-120.25) | 0.514 |
| PLT, *10^9/L | 136 (85.5-220.5) | 154.5 (104.75-229.25) | 121 (78.5-211.5) | 0.193 |
| ALT, U/L | 34 (19-54.5) | 34 (19-76.7) | 29 (19.25-49) | 0.261 |
| TBIL, mmol/L | 13.8 (8.22-30.74) | 15.23 (11.8-39.07) | 11.66 (7.5-27.85) | 0.041^a^ |
| BUN, mmol/L | 8.46 (5.55-13.19) | 9.52 (5.54-11.77) | 7.74 (5.53-14.2) | 0.947 |
| CR, mmol/L | 74.5 (51.83-169.93) | 72.75 (47.25-130.88) | 76.7 (51.83-198.35) | 0.446 |
| CK, umol/L | 130 (54.5-336) | 258 (92.54-656.5) | 85 (36-241) | 0.037^a^ |
| LDH, U/L | 668 (363-956) | 928 (363-1126) | 553.5 (318.25-946.75) | 0.177 |
| GLU, mmol/L | 8.80 (6.56-10.58) | 9.07 (7.59-10.65) | 8.34 (6.19-10.37) | 0.217 |
| ALB, g/L | 30 (27-33.2) | 28.3 (26-32) | 30.45 (27.85-34) | 0.151 |
| K, mmol/L | 4.1 (3.7-4.4) | 4 (3.6-4.5) | 4.2 (3.8-4.4) | 0.326 |
| Na, mmol/L | 139 (135-144) | 141 (136-144) | 138 (135-145.25) | 0.446 |
| BNP, pg/ml | 208.2 (97.74-885.75) | 274.69 (143-1042.25) | 165.8 (80.3-535.4) | 0.037^a^ |
| PCT, ng/ml | 1.92 (0.59-6.96) | 3.4 (0.65-7.12) | 1.46 (0.49-6.85) | 0.362 |
| CRP, mg/L | 71.03 (13.7-192.14) | 56.12 (18.52-200) | 117.11 (8.7-184.27) | 0.911 |
| PT, s | 15.5 (14.3-17.9) | 15.4 (14.7-17.3) | 16 (14.1) | 0.523 |
| APTT, s | 46.4 (38.63-60.20) | 46.1 (38.45-59.75) | 47.3 (38.38-61.25) | 0.996 |
| Fib, g/L | 5.25 (3.07-6.64) | 5.40 (4.25-6.68) | 5.04 (2.52-6.60) | 0.302 |
| D-D, ug/ml | 7.96 (3.49-16.76) | 7.42 (4.27-12.21) | 9.28 (3-20) | 0.868 |
| **Barotrauma during ECMO, number (%)** | 19 (20.9) | 7 (13.2) | 12 (31.6) | 0.033^a^ |

^a^: *P*<0.05; ^b^: *P*<0.001

HFNC, high flow nasal cannula; NPPV, non-invasive positive pressure ventilation; IPPV, invasive positive pressure ventilation; VAP, ventilator associated pneumonia; WBC, white blood cell; LYM, lymphocyte; HB, haemoglobin; PLT, platelet; ALT, alanine transaminase; TBIL, total bilirubin; BUN, blood urea nitrogen; CR, creatinine; CK, creatine kinase; LDH, lactate dehydrogenase; GLU, glucose; ALB, albumin; K, kalium; Na, natrium; BUN, blood urea nitrogen; BNP, brain natriuretic peptide; PCT, procalcitonin; CRP, c-reactive protein; PT, prothrombin time; APTT, activated partial thromboplastin time; D-D, D-dimer; ECMO, extracorporeal membrane oxygenation

Table S2. After propensity score matching, laboratory examinations and respiratory support before ECMO in two groups

|  | ALL  N=50 | Prone group  N=25 | Supine group  N=25 | P |
| --- | --- | --- | --- | --- |
| **Respiratory support** | | | | |
| IPPV, number (%) | 50(100) | 25 (100) | 25 (100) | 1 |
| **Barotrauma before ECMO, number (%)** | 10(20) | 6(24) | 4(16) | 0.480 |
| **VAP, number (%)** | 6(12) | 3(12) | 3(12) | 1 |
| **Tracheotomy, number (%)** | 39(78) | 17(68) | 22(88) | 0.088 |
| WBC, *10^9/L, mean ± SD | 13.08±8.07 | 12.39±7.63 | 13.77±8.60 | 0.560 |
| Lym, *10^9/L, mean ± SD | 0.73±0.48 | 0.65±0.35 | 0.82±0.59 | 0.272 |
| HB, g/L, mean ± SD | 110.04±25.28 | 109.54±21.62 | 110.54±28.95 | 0.893 |
| PLT, *10^9/L, mean ± SD | 178.17±98.70 | 176.46±79.77 | 179.96±117.09 | 0.906 |
| ALT, U/L | 38(23.5-55.5) | 34(24.25-53.0) | 41(22.75-78) | 0.707 |
| TBIL, mmol/L | 14.9(8.57-33.46) | 15.19(11.50-46.29) | 12.90(8.01-31.69) | 0.247 |
| BUN, mmol/L | 7.87(5.45-11.86) | 9.71(5.84-12.13) | 7.24(5.10-12.05) | 0.262 |
| Cr, mmol/L | 73.2(55.15-129.95) | 70.70(53.83-121.95) | 74.20(54.98-169.18) | 0.598 |
| CK, umol/L | 134(82-355) | 199(90.36-379) | 113(34.74-325.75) | 0.277 |
| LDH, U/L | 910(320-1084) | 935(613.5-1531) | 523(262.25-1021.25) | 0.148 |
| GlU, mmol/L, mean ± SD | 9.52±3.32 | 9.84±3.05 | 9.17±3.61 | 0.494 |
| ALB, g/L | 28.5(26-32.3) | 27.35(24.75-30.25) | 30(27.5-34) | 0.052 |
| K, mmol/L, mean ± SD | 4.05±0.52 | 4.02±0.55 | 4.08±0.50 | 0.732 |
| Na, mmol/L, mean ± SD | 140.02±6.74 | 140.04±4.90 | 140.00±8.36 | 0.984 |
| BNP, pg/ml | 188(98.18-761) | 274.69(158-1136) | 101.59(33.98-242.68) | 0.111 |
| PCT, ng/ml | 1.92(0.65-6.23) | 3.27(0.64-6.86) | 1.67(0.77-2.87) | 0.461 |
| CRP, mg/L | 122.63(22.2-200) | 56.12(18.52-200) | 164.07(89.98-200) | 0.428 |
| PT, s | 15.40(14.10-17.30) | 15.4(14.7-17.0) | 14.95(14-18.73) | 0.937 |
| APTT, s | 45.9(36.75-60.2) | 45.9(37.4-60.2) | 46.55(35.48-60.38) | 0.666 |
| Fib, g/L | 5.58(3.74-6.69) | 5.72(4.19-6.67) | 5.30(2.59-6.87) | 0.540 |
| D-D, ug/ml | 8.31(3.54-16.19) | 5.72(4.19-11.76) | 13.07(3.35-20.00) | 0.305 |
| **Barotrauma during ECMO, number (%)** | 9(18) | 5(20) | 4(16) | 1 |

^a^: *P*<0.05; ^b^: *P*<0.01;HFNC, high flow nasal cannula; NPPV, non-invasive positive pressure ventilation; IPPV, invasive positive pressure ventilation; VAP, ventilator associated pneumonia; WBC, white blood cell; Lym, lymphocyte; HB, haemoglobin; PLT, platelet; ALT, alanine transaminase; TBIL, total bilirubin; BUN, blood urea nitrogen; Cr, creatinine; CK, creatine kinase; LDH, lactate dehydrogenase; GLU, glucose; ALB, albumin; K, kalium; Na, natrium; BMI, body mass index; BUN, blood urea nitrogen; BNP, brain natriuretic peptide; PCT, procalcitonin; CRP, c-reactive protein; PT, prothrombin time; APTT, activated partial thromboplastin time; D-D, D-dimer

Table S3. Before propensity score matching, ventilation, ECMO and arterial blood gas parameters before ECMO and the first day of ECMO in two groups

|  | **All**  **N=91** | **Prone group**  **N=38** | **Supine group**  **N=53** | | **P** | |
| --- | --- | --- | --- | --- | --- | --- |
| **Mechanical ventilation parameter before ECMO, IQR** | | | | | |  |
| PC/PS, cmH_2_O | 14 (12-16) | 14 (12-16) | 12 (12-15) | 0.237 | |  |
| PEEP, cmH_2_O | 12 (8-14) | 10 (8-14) | 12 (8-15) | 0.265 | |  |
| Inspiration fraction of oxygen, % | 100 (98.75-100) | 100 (100-100) | 100 (95-100) | 0.809 | |  |
| Respiratory rate, breaths/min | 28 (22.5-33) | 27 (22-33) | 28 (22.75-33.5) | 0.623 | |  |
| Tidal volume, mL | 406 (299-489) | 354.5 (256.75-457.5) | 445 (318.5-533) | 0.123 | |  |
| Pplat, cmH_2_O | 27 (22-31) | 27 (22.25-29.75) | 28 (22-33) | 0.557 | |  |
| **Gas analysis****, IQR** | | | | | |  |
| pH | 7.35 (7.26-7.43) | 7.34 (7.24-7.42) | 7.35 (7.26-7.43) | 0.561 | |  |
| PaO_2_, mmHg | 64.6 (54-79.8) | 65.6 (55.88-76.05) | 63.5 (52.8-82.6) | 0.969 | |  |
| PaO_2_/FiO_2_, mmHg | 64.9 (56-88.7) | 65.9 (55.88-82.60) | 63.3 (56-91.75) | 0.449 | |  |
| PaCO_2_, mmHg | 47.1 (36.15-58.2) | 49.35 (40.75-58.74) | 44.4 (35.2-57.4) | 0.134 | |  |
| Lac, mmol/L | 2.1 (1.5-2.8) | 2.0 (1.5-2.9) | 2.2 (1.5-2.8) | 0.959 | |  |
| **Heart rate, beats/min, IQR** | 120 (103.5-143.5) | 130 (105-145) | 114 (100-143) | 0.467 | |  |
| **Systolic pressure, mmHg, IQR** | 115 (95-130) | 118 (95-130) | 113.5 (94.5-131.25) | 0.671 | |  |
| **Diastolic pressure, mmHg, IQR** | 60 (50-70) | 60 (51-75) | 58 (50-69.25) | 0.229 | |  |
| **Mean arterial pressure, mmHg, IQR** | 77 (66.5-88.5) | 77.5 (68.23-89.25) | 76 (66-86) | 0.521 | |  |
| **Vasoactive drugs, ug/kg/min, IQR** | 0.3 (0.115-0.85) | 0.21 (0.1-0.455) | 0.4 (0.14-1.3) | 0.139 | |  |
| **Mechanical ventilation parameter, the first day of ECMO, IQR** | | | | | |  |
| PC/PS, cmH_2_O | 11 (10-13) | 10 (10-12) | 12 (10-14) | 0.067 | |  |
| PEEP, cmH_2_O | 10 (8-14) | 10.5 (8.5-12) | 10 (7.5-14) | 0.922 | |  |
| Inspiration fraction of oxygen, % | 50 (40-60) | 50 (40-60) | 50 (40-60) | 0.482 | |  |
| Respiratory rate, breaths/min | 20 (15-24) | 20 (15-25) | 19.5 (12.75-23.75) | 0.504 | |  |
| Tidal volume, mL | 238 (144.75-311.75) | 230 (101-291) | 258 (168-328.5) | 0.274 | |  |
| Pplat, cmH_2_O | 22 (20-25) | 22 (20-24) | 23 (19.76-27) | 0.351 | |  |
| **ECMO settings, the first day of ECMO, IQR** | | | | | |  |
| ECMO blood flow, L/min | 4.18 (3.87-4.54) | 4.25 (3.95-4.71) | 4.09 (3.72-4.50) | 0.194 | |  |
| Sweep gas flow, L/min | 4 (3.5-5.0) | 5 (4-6) | 4 (3-5) | 0.047 | |  |
| Membrane lung fraction of oxygen, % | 100 (100-100) | 100 (100-100) | 100 (100-100) | 0.434 | |  |
| **Gas analysis, IQR** | | | | | |  |
| pH | 7.42 (7.36-7.46) | 7.42 (7.36-7.44) | 7.42 (7.35-7.46) | 0.979 | |  |
| PaO_2_ | 73.2 (63.6-86.45) | 72.4 (62.3-85.65) | 76.95 (64.45-90.88) | 0.547 | |  |
| PaO_2_/FiO_2_ | 148 (84.25-191.25) | 144 (90.9-179.5) | 151.5(77.69-234.88) | 0.974 | |  |
| PaCO_2_ | 42.9 (39.45-47.6) | 44.3 (40.55-48.25) | 41.55 (39.03-46.83) | 0.205 | |  |
| Lac | 2.1 (1.5-3.6) | 2.1 (1.5-3.65) | 2.1 (1.4-3.4) | 0.914 | |  |
| **Heart rate, beats/min, IQR** | 100 (80-120) | 100 (79-127.5) | 94.5 (81.25-113) | 0.336 | |  |
| **Systolic pressure, mmHg, IQR** | 126 (110-142) | 126.5 (110-139.5) | 126 (110-145) | 0.858 | |  |
| **Diastolic pressure, mmHg, IQR** | 63 (55-75) | 62.5 (52.25-75.75) | 63 (55-74) | 0.807 | |  |
| **Mean arterial pressure, mmHg, IQR** | 81 (73.65-95) | 87 (75-97) | 80 (71.75-95) | 0.239 | |  |
| **Vasoactive drugs, ug/kg/min, IQR** | 0.3 (0.1-1.0) | 0.21 (0.09-0.98) | 0.44 (0.11-1.4) | 0.387 | |  |

^a^: P<0.05; ^b^: P<0.01; PC, pressure control; PS, pressure support; PEEP, positive end expiratory pressure; pH, potential of hydrogen; PaO_2_, arterial oxygen pressure; FiO_2_, fraction of inspiration; PaCO_2_, partial pressure of arterial carbon dioxide; Lac, lactic acid; ECMO, extracorporeal membrane oxygenation.

Table S4. After propensity score matching, ventilation, ECMO and arterial blood gas parameters before ECMO and the first day of ECMO in two groups

|  | | **ALL**  **N=50** | **Prone group**  **N=25** | **Supine group**  **N=25** | **P** |
| --- | --- | --- | --- | --- | --- |
| **Mechanical ventilation parameter before ECMO** | | | | | |
| PC/PS(cmH_2_O) | 14(12-16) | | 14(12-15.75) | 14(12-16) | 0.980 |
| PEEP (cmH_2_O) | 12(10-14) | | 11(10-14) | 12(8-15) | 0.434 |
| Inspiration fraction of oxygen (%) | 100(97.5-100) | | 100(85-100) | 100(97.5-100) | 0.848 |
| Respiratory rate (breaths/min) | 28.95±7.66 | | 28.43±8.02 | 29.52±7.40 | 0.642 |
| Tidal volume (mL/kg) | 5.85(4.42-7.54) | | 4.97(4.11-6.84) | 6.83(4.46-8.87) | 0.239 |
| Ppeak (cmH_2_O) | 27(22-30) | | 27(22-28) | 29(22-31) | 0.400 |
| **Gas analysis** | | | | | |
| pH | 7.38(7.25-7.43) | | 7.39(7.25-7.43) | 7.38(7.25-7.43) | 0.676 |
| PaO_2_ (mmHg) | 63.95(52.80-75.93) | | 65.5(60-75.4) | 60.7(50.7-76.9) | 0.362 |
| PaO_2_/FiO_2_ (mmHg) | 64.95(56.00-92.90) | | 65.70(60.60-84.00) | 63(48.65-95.35) | 0.810 |
| PaCO_2_ (mmHg) | 47.05(39.78-58.67) | | 49.2(40-57.4) | 47(39.1-65) | 0.775 |
| Lac (mmol/L) | 2.10(1.50-2.60) | | 1.95(1.53-2.55) | 2.2(1.5-2.8) | 0.709 |
| **Heart rate, beats/min** | 126.70±26.54 | | 125.58±28.01 | 127.91±25.44 | 0.770 |
| **Systolic pressure, mmHg** | 117±27.30 | | 117.65±29.58 | 116.32±25.37 | 0.872 |
| **Diastolic pressure, mmHg** | 61.24±13.83 | | 63.65±14.98 | 58.73±12.37 | 0.237 |
| **Vasoactive drugs, ug/kg/min** | 0.30(0.16-0.88) | | 0.25(0.12-0.43) | 0.35(0.20-1.30) | 0.260 |
| **Mechanical ventilation parameter, the first day of ECMO** | | | | | |
| PC/PS (cmH_2_O) | 10(10-12) | | 10(10-12) | 11.5(10-14) | 0.361 |
| PEEP (cmH_2_O) | 12(8-13) | | 10(8-12) | 12(7.5-14) | 0.374 |
| Inspiration fraction of oxygen (%) | 50(40-60) | | 50(41.25-60) | 45(40-60) | 0.419 |
| Respiratory rate (breaths/min) | 18.89±6.56 | | 20.28±7.09 | 17.24±5.59 | 0.118 |
| Tidal volume (mL/kg) | 3.35(2.15-4.79) | | 3.35(1.99-4.71) | 3.60(2.40-5.04) | 0.497 |
| Ppeak (cmH_2_O) | 22(20-25) | | 22(19-24) | 24(20-27) | 0.667 |
| **ECMO settings, the first day of ECMO** | | | | | |
| ECMO blood flow (L/min) | 4.16±0.61 | | 4.20±0.64 | 4.11±0.58 | 0.624 |
| Sweep gas flow (L/min) | 4.0(3.5-5.1) | | 4.5(3.1-6.0) | 4(3.75-5) | 0.653 |
| Membrane lung fraction of oxygen (%) | 100(100-100) | | 100(100-100) | 100(97.5-100) | 0.063 |
| **Gas analysis** | | | | | |
| pH | 7.42(7.36-7.45) | | 7.42(7.36-7.44) | 7.42(7.36-7.46) | 0.893 |
| PaO_2_ | 71.90(61.40-84.30) | | 71.65(61.85-75.40) | 71.9(60.2-83.8) | 0.856 |
| PaO_2_/FiO_2_ | 90.90(73.08-174.00) | | 99.4(71.45-178.25) | 79.75(66.28-171.56) | 0.562 |
| PaCO_2_ | 43.00(40.20-46.90) | | 44.02(40.53-47.43) | 42(39.5-46.6) | 0.558 |
| Lac | 2.00(1.40-3.00) | | 2.05(1.53-2.95) | 1.9(1.2-3.2) | 0.717 |
| **Heart rate, beats/min** | 99.54±22.13 | | 99.44±22.16 | 99.65±22.59 | 0.974 |
| **Systolic pressure, mmHg** | 125.13±22.00 | | 129.25±18.88 | 120.83±24.44 | 0.192 |
| **Diastolic pressure, mmHg** | 62.87±14.23 | | 64.42±15.42 | 61.26±13.03 | 0.453 |
| **Vasoactive drugs, ug/kg/min** | 0.23(0.10-0.90) | | 0.2(0.09-1.22) | 0.24(0.10-0.55) | 0.832 |

^a^: P<0.05; ^b^: P<0.01; PC, pressure control; PS, pressure support; PEEP, positive end expiratory pressure; pH, potential of hydrogen; PaO_2_, arterial oxygen pressure; FiO_2_, fraction of inspiration; PaCO_2_, partial pressure of arterial carbon dioxide; Lac, lactic acid; ECMO, extracorporeal membrane oxygenation.

Table S5. Complication of ECMO

|  | **Prone Group(n=38)** | **Supine Group(n=53)** | ***p*** |
| --- | --- | --- | --- |
| **Oxygenation dysfunction** | 2(5.3) | 2(3.8) | 1 |
| **Thrombosis** | 4(10.5) | 10(18.9) | 0.277 |
| **Haemorrhagic** | 19(50.0) | 21(39.6) | 0.325 |
| **Renal** | 20(52.6) | 31(58.5) | 0.579 |
| **Neurologic** | 3(8.8) | 12(25.5) | 0.056 |
| **Cardiovascular** | 15(39.5) | 28(52.8) | 0.208 |
| **Pulmonary** | 8(21.1) | 7(13.2) | 0.320 |

Table S6. A multivariable logistic regression of ECMO weaning rate

|  | **OR(95% CI)** | ***P*** |
| --- | --- | --- |
| **Prone position during ECMO** | 1.836 (0.615-5.479) | 0.276 |
| **Age** | 0.983 (0.953-1.013) | 0.265 |
| **Pathogen spectrum(Viral)** | 0.903 (0.332-2.458) | 0.842 |
| **Respiratory support (IPPV)** | 0.510 (0.109-2.386) | 0.392 |
| **Barotrauma before ECMO** | 0.760 (0.217-2.662) | 0.668 |
| **Barotrauma during ECMO** | 1.084 (0.312-3.767) | 0.900 |
| **Prone position before ECMO** | 0.996 (0.374-2.652) | 0.994 |
| **PaO2/FiO2 ratio prior to ECMO** | 0.992 (0.980-1.005) | 0.245 |

ECMO, extracorporeal membrane oxygenation; IPPV, invasive positive pressure ventilation

Table S7. A multivariable logistic regression of ICU survival

|  | **OR(95% CI)** | **P** |
| --- | --- | --- |
| **Prone position during ECMO** | 1.282 (0.399-4.116) | 0.677 |
| **Age** | 0.968 (0.936-1.001) | 0.057 |
| **Pathogen spectrum(Viral)** | 1.697 (0.577-4.988) | 0.336 |
| **Respiratory support (IPPV)** | 0.264 (0.053-1.309) | 0.103 |
| **Barotrauma before ECMO** | 0.972 (0.264-3.582) | 0.966 |
| **Barotrauma during ECMO** | 0.508 (0.122-2.108) | 0.351 |
| **Prone position before ECMO** | 0.535 (0.182-1.570) | 0.255 |
| **PaO2/FiO2 ratio prior to ECMO** | 0.996 (0.983-1.009) | 0.520 |

ECMO, extracorporeal membrane oxygenation; IPPV, invasive positive pressure ventilation

Table S8. A multivariable logistic regression of hospital survival

|  | **OR(95% CI)** | **P** |
| --- | --- | --- |
| **Prone position during ECMO** | 1.620 (0.464-5.645) | 0.449 |
| **Age** | 0.961 (0.926-0.996) | 0.030 |
| **Pathogen spectrum(Viral)** | 1.917 (0.592-6.215) | 0.278 |
| **Respiratory support (IPPV)** | 0.211 (0.036-1.221) | 0.082 |
| **Barotrauma before ECMO** | 1.080 (0.276-4.231) | 0.912 |
| **Barotrauma during ECMO** | 0.581 (0.127-2.667) | 0.485 |
| **Prone position before ECMO** | 0.555 (0.171-1.798) | 0.326 |
| **PaO2/FiO2 ratio prior to ECMO** | 0.980 (0.957-1.003) | 0.082 |

ECMO, extracorporeal membrane oxygenation; IPPV, invasive positive pressure ventilation

Table S9. The medium number of pronation for every patients

| **Patients** | **The number of pronation** |
| --- | --- |
| 1 | 5 |
| 2 | 5 |
| 3 | 7 |
| 4 | 4 |
| 5 | 6 |
| 6 | 1 |
| 7 | 1 |
| 8 | 5 |
| 9 | 2 |
| 10 | 4 |
| 11 | 4 |
| 12 | 4 |
| 13 | 1 |
| 14 | 2 |
| 15 | 1 |
| 16 | 5 |
| 17 | 1 |
| 18 | 4 |
| 19 | 7 |
| 20 | 5 |
| 21 | 4 |
| 22 | 8 |
| 23 | 3 |
| 24 | 15 |
| 25 | 3 |
| 26 | 1 |
| 27 | 11 |
| 28 | 3 |
| 29 | 1 |
| 30 | 7 |
| 31 | 2 |
| 32 | 3 |
| 33 | 3 |
| 34 | 2 |
| 35 | 10 |
| 36 | 5 |
| 37 | 3 |
| 38 | 9 |

Table S10. STROBE Statement—Checklist of items that should be included in reports of cohort studies

**Item**

**No Recommendation**

| **Title and abstract** | 1 | (*a*) Indicate the study’s design with a commonly used term in the title or the abstract (*b*) Provide in the abstract an informative and balanced summary of what was done and what was found |
| --- | --- | --- |
| **Introduction** |  |  |
| Background/rationale | 2 | Explain the scientific background and rationale for the investigation being reported |
| Objectives | 3 | State specific objectives, including any prespecified hypotheses |
| **Methods** |  |  |
| Study design | 4 | Present key elements of study design early in the paper |
| Setting | 5 | Describe the setting, locations, and relevant dates, including periods of recruitment, exposure, follow-up, and data collection |

Participants 6 (*a*) Give the eligibility criteria, and the sources and methods of selection of participants. Describe methods of follow-up

|  |  | (*b*) For matched studies, give matching criteria and number of exposed and unexposed |
| --- | --- | --- |
| Variables | 7 | Clearly define all outcomes, exposures, predictors, potential confounders, and effect modifiers. Give diagnostic criteria, if applicable |
| Data sources/ measurement | 8* | For each variable of interest, give sources of data and details of methods of assessment (measurement). Describe comparability of assessment methods if there is more than one group |
| Bias | 9 | Describe any efforts to address potential sources of bias |
| Study size | 10 | Explain how the study size was arrived at |
| Quantitative variables | 11 | Explain how quantitative variables were handled in the analyses. If applicable, describe which groupings were chosen and why |

Statistical methods 12 (*a*) Describe all statistical methods, including those used to control for confounding

1. Describe any methods used to examine subgroups and interactions
2. Explain how missing data were addressed
3. If applicable, explain how loss to follow-up was addressed

|  |  | (*e*) Describe any sensitivity analyses |
| --- | --- | --- |
| **Results** |  |  |

Participants 13* (a) Report numbers of individuals at each stage of study—eg numbers potentially eligible, examined for eligibility, confirmed eligible, included in the study, completing follow-up, and analysed

(

b) Give reasons for non-participation at each stage

(

c) Consider use of a flow diagram

Descriptive data 14* (a) Give characteristics of study participants (eg demographic, clinical, social) and information on exposures and potential confounders

1. Indicate number of participants with missing data for each variable of interest
2. Summarise follow-up time (eg, average and total amount)

Outcome data 15* Report numbers of outcome events or summary measures over time

Main results 16 (*a*) Give unadjusted estimates and, if applicable, confounder-adjusted estimates and their precision (eg, 95% confidence interval). Make clear which confounders were adjusted for and why they were included

(*b*) Report category boundaries when continuous variables were categorized

|  |  | (*c*) If relevant, consider translating estimates of relative risk into absolute risk for a meaningful time period |
| --- | --- | --- |

1

| Other analyses 17 | Report other analyses done—eg analyses of subgroups and interactions, and sensitivity analyses |
| --- | --- |
| **Discussion** |  |
| Key results 18 | Summarise key results with reference to study objectives |
| Limitations 19 | Discuss limitations of the study, taking into account sources of potential bias or imprecision. Discuss both direction and magnitude of any potential bias |
| Interpretation 20 | Give a cautious overall interpretation of results considering objectives, limitations, multiplicity of analyses, results from similar studies, and other relevant evidence |
| Generalisability 21 | Discuss the generalisability (external validity) of the study results |
| **Other information** |  |

Funding 22 Give the source of funding and the role of the funders for the present study and, if applicable, for the original study on which the present article is based

*Give information separately for exposed and unexposed groups.

2
